# Ethical challenges in conducting maternal-fetal surgery trials. A systematic review

**DOI:** 10.1038/s41390-024-03734-y

**Published:** 2024-12-05

**Authors:** Alice Cavolo, Chris Gastmans, Neeltje Crombag

**Affiliations:** 1 Institute of Biomedical Ethics and History of Medicine (IBME), University of Zurich, Zurich, Switzerland; 2 Centre for Biomedical Ethics and Law, KU Leuven, Leuven, Belgium; 3 Department of Development and Regeneration Cluster Woman and Child, KU Leuven, Leuven, Belgium; 4 Divisie Vrouw en Baby, Universitair Medisch Centrum Utrecht, Utrecht, The Netherlands

## Abstract

**Objective:**

To present the ethical challenges embedded in published maternal-fetal surgery (MFS) trials and their potential solutions.

**Method:**

Systematic review of normative and empirical literature. We selected articles based on predefined inclusion criteria. QUAGOL methodology was used for analysis.

**Results:**

Forty-three articles were included. We identified two main themes. First, clinical ethics issues. One of the main challenges is balancing rights of the fetus with the rights of the pregnant person. There seems to be an agreement that the pregnant person has the right to decide whether to participate regardless of fetal benefit. Second, research ethics issues. The main issues are difficulties in obtaining a sizeable sample, which lead to trials delays and cancellations, and in obtaining appropriate standardization. These difficulties have important ethical ramifications. For example, trial cancellations due the lack of proper sample size generate a waste of resources and pointlessly place participants at risk as conclusive evidence on MFS efficacy was not obtained.

**Conclusions:**

We need to develop creative solutions that can prevent some of these ethical concerns. We need to involve all the relevant stakeholders in the development process. Further, researchers should discuss what practical issues they encountered and how they addressed them in their publications.

**Impact statement:**

- Understanding the ethical challenges embedded in MFS trials will help improving future trials and, consequently, clinical outcomes.
- To our knowledge, this is the first systematic review unveiling the ethical challenges in maternal-fetal surgery trials. To offer a complete overview of the challenges, we included both normative and empirical literature.
- We found that the main ethical challenges are practical difficulties that have important ethical ramification. E.g., difficulties in recruitment might hinder scientific validity, which in turn might lead to suboptimal treatment.

## Introduction

Over the past decades, modern medicine achieved important results in the field of fetal diagnostics and treatment that led to improvements in survival and quality of life. However, as all fetal medicine inevitably involves not only the fetus but also the pregnant person, it presents healthcare providers with new challenges, such as how to balance the interests of the pregnant person with the interests of the fetus/future infant.^1^

Within the broader field of fetal medicine, maternal fetal surgery (MFS) [We decided to adopt the term “maternal-fetal” instead of “fetal” surgery to emphasize that the pregnant person is also a research participant in accordance with Chervenak and McCullough ^2^ and Crombag, Pizzolato et al.^3^] trials are amongst the most challenging scenarios.^4^ On the one hand, these trials have the potential to improve survival and quality of life of infants with various congenital anomalies. The MOMS trial for example demonstrated that prenatal repair of spina bifida is safer for the fetus and leads to better outcomes than the postnatal repair.^5^ On the other hand, these trials require an invasive surgery on two participants, the pregnant person and the fetus, whose interests do not necessarily coincide.^6^ This raises important ethical questions. To what extent can we justify an experimental invasive procedure with significant risks on the pregnant persons’ body for fetal benefit? And how do we ensure an authentic informed consent? Further, MFS trials can also be challenging regarding the design and methodology. For example, congenital anomalies requiring MFS are often rare hindering the recruitment and the success of the trial.^7^

A better understanding of the ethical issues raised by MFS trials is necessary to improve future trials and adequately address these issues. Although much has been written on the ethics of MFS trials, both in normative and empirical literature, to our knowledge no review has collected and summarized this knowledge yet, meaning that a comprehensive understanding of these issues is still lacking. Hence, our review aims at identifying ethical issues raised by MFS trials and possible solutions as described by the normative and empirical literature.

## Methodology

We conducted a systematic review of empirical and theoretical literature on MFS trials.^8,9^ The review protocol was registered in Open Science Framework. [The review does not have a number but it can be accessed on the website of Open Science Framework using the review title or the following link: https://osf.io/xw9jy/] A systematic search of Medline®, Embase®, Web of Science™, CINAHL and PhiliPapers electronic databases was conducted on January 18th 2024. Search strings consisted of three categories of words, one identifying the MFS trials (e.g., MOMS trial, TOTAL trial), one focusing on the MFS (e.g., fetal therapies, fetal surgery), and another focusing on clinical trials (e.g., trial, randomized controlled trial) (Additional file 1). A librarian from the Learning Center Désiré Collen (KU Leuven) assisted with the development of the search string. Results were merged and duplicates were deleted before selecting eligible articles.

**Fig. 1:**
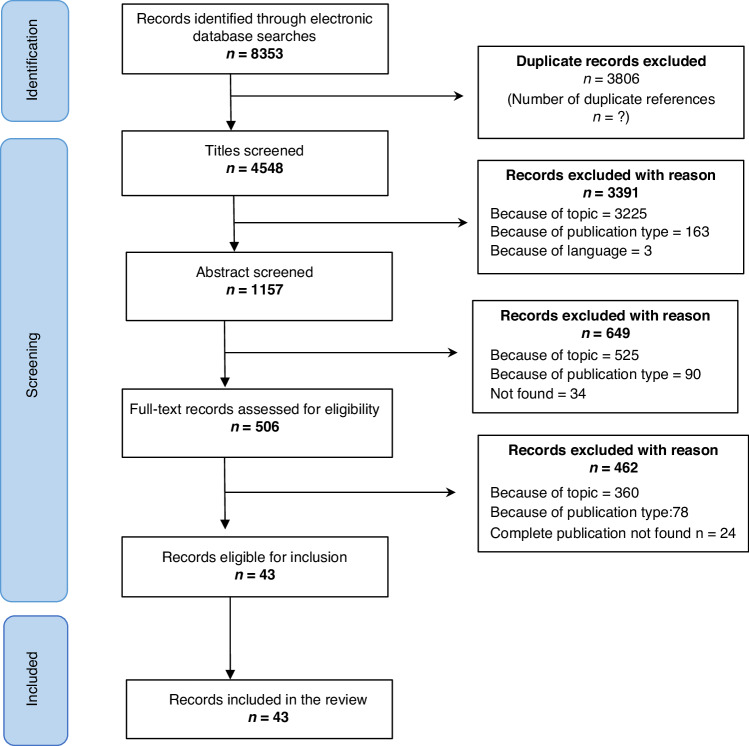
PRISMA flowchart illustrating the process for identifying and selecting articles. The flowchart is organized according to PRISMA guidelines outlined in Liberati et al. Page 4

Eligible articles were selected based on predefined inclusion/exclusion criteria (Table 1). Two authors (AC and NC) independently screened titles, abstracts, and full texts using Rayyan. Disagreements about inclusion were resolved by discussion until consensus was reached. A PRISMA flow diagram^10^ summarizes the literature search process (Fig. 1).

**Table 1:** Inclusion and exclusion criteria for the selection of articles

	Included	Excluded
Types of publication	• Published articles, including normative and empirical articles. • Publications describing clinical trials are eligible if they contain ethical argumentations and only data related to ethical argumentations will be extracted. • Editorials. • Letters to the editors.	• Systematic and narrative reviews because reviewed articles are already included with the risk of duplicating results and over-emphasizing certain positions. • Dissertations, books, book chapters, guidelines, ethics policies and codes, because these publications cannot be systematically searched, which will affect the reproducibility.
Topic	• Publications focusing on maternal-fetal surgery trials.	• Publications on non-surgical maternal-fetal trials, e.g., pharmaceutical trials. • Publications on maternal-fetal medicine in general that does not contain arguments specifically related to maternal-fetal surgery trials. • Publications describing clinical trials or the technical surgical procedures without ethical reflection. • Publications describing legal regulations without ethical reflection.
Language	• Publication language is English.	• Non-English language publications.
Date	• Screening of articles was not limited by publication date; the entire date range was included in searches of Medline, Embase, Web of Science, CINAHL, and PhilPapers databases.	• NA

For quality appraisal, we used the tool developed by Hawker et al.^11^ for quantitative studies, and the CASP scale for qualitative studies.^12^ (Table 2). As there is no standard for assessing argument-based literature,^13^ we relied on the journals’ peer review process to assume that the quality of included articles was sufficient. This is acceptable as the aim of our review is descriptive not normative.^13^

**Table 2:** Quality appraisal of quantitative and qualitative studies using the appraisal criteria of Hawker et al.^a^ and CASP^b^

Hawker et al. Criteria^a^ for quantitative studies (*n* = 5)											
Study	(1) Abstract and Title	(2) Introduction and Aims	(3) Method and Data	(4) Sampling	(5) Data Analysis	(6) Ethics and Bias	(7) Results	(8) Transferability or Generalizability	(9) Implications and Usefulness	Overall Assessment (out of 36 possible)	
Engels et al. ^35^	Good	Good	Good	Good	Good	Fair	Good	Good	Good	High	
Lyerly et al. ^22^	Good	Good	Fair	Fair	Poor	Fair	Good	Good	Fair	Moderate	
Sanz Cortes et al. ^44^	Good	Good	Good	Good	Good	Fair	Good	Good	Good	High	
Vergote et al. 2020	Good	Good	Good	Good	Good	Good	Good	Good	Good	High	
Vergote et al. ^50^	Good	Good	Good	Good	Good	Good	Good	Good	Good	High	
CASP Criteria^b^ for qualitative studies (*n* = 1)											
Study	(1) Clear statement of aims?	(2) Appropriate methodology?	(3) Appropriate design?	(4) Appropriate recruitment strategy?	(5) Appropriate data collection?	(6) Adequate consideration of the relationship between researcher and participants?	(7) Ethical issues considered?	(8) Data analysis sufficiently rigorous?	(9) Clear statement of findings?	(10) Was the research valuable?	Overall assessment (out of 30 possible)
Rothschild et al. ^32^	Yes	Yes	Yes	Unclear	Yes	Yes	Yes	Unclear	Yes	Yes	High

*CASP* Critical Appraisal Skills Program.

^a^Hawker et al.^24^ appraisal questions: (1) Did they provide a clear description of the study? (2) Was there a clear background and a clear statement of the aims of the research? (3) Is the method appropriate and clearly explained? (4) Was the sampling strategy appropriate to address the aims? (5) Was the description of the data analysis sufficiently rigorous? (6) Have ethical issues been addressed and what has necessary ethical approval gained? (7) Is there a clear statement of the findings? (8) Are the findings of this study transferable (generalizable) to a wider population? (9) How important are these findings for policies and practice?

Scoring: Good = 4; Fair = 3; Poor = 2; Very poor = 1.

^b^CASP^25^ appraisal questions: (1) Was there a clear statement of the aims of the research? (2) Was a qualitative methodology appropriate? (3) Was the research design appropriate to address the aims? (4) Was the recruitment strategy appropriate to the aims? (5) Were the data collected in a way that addressed the research issue? (6) Was the relationship between researcher and participants adequately considered? (7) Were ethical issues taken into consideration? (8) Was the data analysis sufficiently rigorous? (9) Was there a clear statement of findings? (10) How valuable was the research? Scoring: Yes=3; Unclear=2; No=1.

Data analysis and synthesis followed the Qualitative Analysis Guide of Leuven (Additional file 2).^14,15^ This analytic method is divided into four steps. First, we repeatedly read the materials to familiarize ourselves with the original sources, Second, we developed conceptual schemes for each individual articles that summarized the main concepts emerging from the articles in answer to the research question. Third, we developed a general conceptual scheme that merged and organized to content of the individual schemes. Finally, following the structure provided by the general scheme, we reported the results. The analysis was conducted by an interdisciplinary research team comprising expertise in maternal-fetal medicine (NC) and bioethics (AC, CG).

## Results

We included 43 articles, six empirical studies and 37 theoretical articles. Most empirical studies (5/6) were high quality, and one was moderate (Table 2). All included articles originated from high-income western countries, mainly from the USA (*n* = 30) and Belgium (*n* = 6). Nineteen articles discuss ethical issues of all MFS trials, while a minority of articles discuss ethical issues of specific MFS trials, mainly the TOTAL trial for congenital diaphragmatic hernia (*n* = 9), the MOMS trial for myelomeningocele (*n* = 8), and the PLUTO trial for lower urinary tract obstruction (*n* = 2). Detailed information on the characteristics of included articles is described in Table 3.

**Table 3:** Characteristics of included articles

Article	Aim	Type of article	Trial	Condition	First author’s country	First author’s profession
Antiel and Flake ^47^	To explore the main challenges of MFS trials.	Theoretical/Normative	Generic	Not specified	USA	Clinical doctor
Bartlett and Bliton ^29^	To explore the main challenges of MFS trials	Commentary; Reply to Radic et al.^24^	MOMS study	Spina bifida; Myelomeningocele	USA	Professor/researcher
Bartlett et al.^30^	To discuss consent in MFS trials.	Commentary	MOMS study	Spina bifida; Myelomeningocele	USA	Professor/researcher
Bliton ^28^	To understand hope in ethics consultation for MFS.	Theoretical/descriptive (based on empirical study)	MOMS study	Spina bifida; Myelomeningocele	USA	Clinical ethics consultant
Cass ^27^	To report the experience with trials for CDH	Theoretical/descriptive (based on trial experience)	TOTAL trial	Congenital diaphragmatic hernia	USA	Clinical doctor
Chervenak and McCullough^2^	To identify the ethical criteria for fetal research	Theoretical/normative	Generic	Not specified	USA	Clinical doctor
Chervenak and McCullough ^4^	To provide an ethical framework for fetal research	Theoretical/normative	Generic	Not specified	USA	Clinical doctor
Chervenak et al. ^17^	To identify the ethical criteria for fetal research	Theoretical/normative	Generic	Not specified	USA	Clinical doctor
Chervenak and McCullough 2011	To develop an ethically justified framework for clinical investigation with pregnant and fetal patients	Theoretical/normative	Generic	Not specified	USA	Clinical doctor
Chervenak and McCullough^31^	To identify the ethical criteria for fetal research	Theoretical/normative	Generic	Generic; Spina bifida	USA	Clinical doctor
Chescheir and D’Alton^39^	To involve obstetricians in the recruitment for the MOMS trial; To develop the science informing daily practice	Commentary	MOMS study	Myelomeningocele	USA	Professor/clinical doctor
Chescheir and Socol^38^	To report a workshop discussion and provide advice for MFS trials	Commentary	MOMS study	Myelomeningocele	USA	Professor/clinical doctor
Crombag et al. ^49^	To provide ethical reflection on whether FETO should be provided also off trial	Theoretical/normative	TOTAL trial	Congenital diaphragmatic hernia	Belgium	Professor/clinical doctor
Crombag et al. ^3^	To respond to Hendriks et al. ^16^	Commentary; Response to Hendriks et al.^16^	Generic	Not specified	Belgium	Professor/clinical doctor
De Bie et al.^26^	To review and discuss challenges of MFS	Theoretical/case analysis	/	Congenital diaphragmatic hernia	Belgium	Clinical doctor
Engels et al.^35^	To understand whether website-provided information about CDH and fetal therapy provides added value.	Empirical/quantitative	TOTAL trial	Congenital diaphragmatic hernia	Belgium	Clinical doctor/clinical research fellow
Fisk et al. ^51^	To discuss the current status of TTS and its possible management	Theoretical	Eurofetus trial	Twin-to-twin transfusion syndrome	Australia	Researcher
Flake ^36^	To discuss the ethical consideration for MFS	Theoretical/normative	Generic	Not specified	USA	Clinical doctor
Harrison ^34^	To address (1) What diseases are treatable and how have we done in treating them to date (2) Who is the fetus’s doctor (3) How should the care of the fetus be organized?	Theoretical/normative	Generic	Not specified	USA	Clinical doctor
Hendriks et al. ^25^A	To develop an ethical framework to identify and weigh risks and benefits of MFS	Theoretical/normative	Generic	Not specified	USA	Researcher
Hendriks et al. ^16^B	To develop an ethical framework to weigh risks and benefits of MFS	Theoretical/normative	Generic	Not specified	USA	Researcher
Lally et al. ^40^	To understand the role of multi-institutional collaboration and patient registries	Theoretical/normative	Not specified	Congenital diaphragmatic hernia	USA	Database coordinator
Laskay et al.^48^	To determine the generalizability of MOMS results and compare shunt rates.	Theoretical/Normative	MOMS study	Myelomeningocele	USA	Clinical researcher
Lyerly and Mahowald^22^	To address the need to consider the pregnant person as research subject using equipoise	Theoretical/normative	Generic	Not specified	USA	Clinical doctor
Lyerly and Mahowald^19^	To address Who is the patient? And, how (if at all) is it possible to do ethically appropriate research on the procedure?	Theoretical/normative	Generic	Myelomeningocele	USA	Clinical doctor
Lyerly et al. ^22^A	To understand the attitudes of members of the Society for Maternal-Fetal Medicine regarding the clinical, scientific, ethical, and policy issues in maternal-fetal surgery.	Empirical/quantitative	Generic	Non-lethal conditions	USA	Clinical doctor
Lyerly et al.^23^B	To understand the ethical issues raised in maternal fetal surgery for non-lethal conditions	Theoretical/normative	Generic	Non-lethal conditions	USA	Clinical doctor
Lyerly^6^		Commentary; Reply to Chervenack & McCullough 2003	Generic	Spina bifida	USA	Clinical doctor
Lyerly et al. ^60^		Commentary; Reply to Hendriks et al.^16^	Generic	Not specified	USA	Clinical doctor
Marquart et al.^46^	To discuss the history, current management, and controversies surrounding surgical intervention for Myelomeningocele	Theoretical/normative	MOMS study	Myelomeningocele	USA	Clinical doctor
Morris et al.^33^	To highlight difficulties of MFS trials based on their own experience	Theoretical/descriptive	PLUTO	Congenital lower urinary tract obstruction	UK	Professor/researcher
Premkumar et al.^42^	To understand the value and limitation of using predefined eligibility criteria in trials and clinical implementation	Theoretical/normative	MOMS study	Myelomeningocele	USA	Clinical doctor/professor
Radic et al.^24^	To address ethical issues raised by MFS in trial and implementation	Theoretical/normative	Generic	Not specified	Canada	Clinical doctor/professor
Reitsma and Moreno^20^	To analyze ethical issues in MFS and the related regulations	Theoretical/normative	Generic	Not specified	USA	Clinical doctor/professor
Rodrigues and Van Den Berg^21^	To understand why equipoise is unsuitable for MFS trials	Theoretical/normative	TOTAL trial	Congenital diaphragmatic hernia	The Netherlands	Legal advisor/researcher
Rodrigues et al. ^43^	To understand whether RCTs are adequate for the clinical evaluation of maternal–fetal surgery for congenital diaphragmatic hernia	Theoretical/normative	TOTAL trial	Congenital diaphragmatic hernia	The Netherlands	Legal advisor/researcher
Rothschild et al.^32^	To explore the cultural and life experiential foundations of the decision-making process of the participants in a feasibility trial for maternal–fetal surgery for myelomeningocele.	Empirical/qualitative	Feasibility trial for in utero surgery for neural tube defects	Spina bifida, Myelomeningocele	USA	Professor/researcher
Sanz Cortes et al. ^44^	To report the main outcomes in the first 12 months of life of children undergoing prenatal fetoscopic repair of open spina bifida included in an international registry and to compare these with the results reported in the MOMS study	Trial report		Spina bifida, Myelomeningocele	USA	Clinical doctor
Stolar et al.^45^		Commentary; Reply to Deprest et al. 2021	TOTAL trial	Congenital diaphragmatic hernia	USA	Clinical doctor
Van Mieghem and Rayan^7^	To discuss the issues related to the PLUTO trial	Commentary	PLUTO trial	Lower urinary tract obstruction	Canada	Clinical doctor
Vergote et al. 2020	To assess the knowledge, attitude and practice of maternal-fetal medicine specialists toward the antenatal management of CDH, and the TOTAL trial	Empirical/quantitative	TOTAL trial	Congenital diaphragmatic hernia	Belgium	Clinical doctor
Vergote et al.^50^	To explore the views and practices of maternal‐fetal medicine specialists on offering FETO after the TOTAL trial	Empirical/quantitative	TOTAL trial	Congenital diaphragmatic hernia	Belgium	Clinical doctor
Ville ^41^	To argue that FETO centers should make their own decisions, based on their own results and the individual patient’s utility choices, on whether to offer FETO outside the trial.	Commentary	TOTAL trial	Congenital diaphragmatic hernia	France	Clinical doctor

We identified two main themes: clinical ethics issues and solutions (i.e., coexistence of two participants, risk and benefits assessments, and informed consent) and research ethics issues and solutions (i.e., randomization, generalizability, and equipoise)

### Clinical-ethics issues and solutions

#### The existence of two participants: the pregnant person and the fetus

Six publications identified the coexistence of the two participants and the risk-benefit asymmetry as the main clinical-ethical issue of MFS trials.^2,4,16–19^ That is because although MF surgeries are operated on the pregnant person they are mainly done for fetal benefit and, although the interests of pregnant persons and fetuses are intertwined, they are not always aligned and sometimes even in conflict, which complicates the risk-benefit assessment. We identified two main conceptual solutions to this issue.

The first solution is to conceptualize the fetus as a patient.^2,4,17,18^ According to this position, the fetus does not have independent moral status, rather its moral status depends on the pregnant person’s decision to continue the pregnancy and to present the fetus to the clinician as a patient for care. Once the fetus becomes a patient, then the clinician has beneficence obligations towards it, meaning that MFS is justifiable. However, beneficence obligations toward the fetus “must, in all cases, be considered along with beneficence-based and autonomy-based obligations to the pregnant woman.”^14, p.11]^

The second solution is to conceptualize the pregnant person as the sole or primary patient.^6,20–23^ According to this position, as pregnant people are autonomous patients, they have the right to decide for themselves whether the benefits of the MFS trial outweigh the risks. Hence, although the partner has an interest in the care of the fetus, the pregnant person’s decision supersedes the interests of the fetus and the opinion of the partner as the operation is physically conducted on their body.^6,20–22,24^

#### Risk-benefit assessment

Risks-benefits assessments are particularly tricky in MFS trials due to the above-mentioned presence of two participants and of the asymmetry in the distribution of risks and benefits.^2,4,16–19^

When it comes to the fetus, Hendriks et al. explained that many prenatal surgeries are less risky than the postnatal surgery, making the MFS trial generally more beneficial for the fetus, position also assumed by others.^6,16,19,22,25^ Nevertheless, five publications emphasize that fetal risks should be minimized^2,3,16,18,26^ and one maintained that only fetuses with less than 25% chances of survival should be included in the trial.^27^ De Bie et al. also reminded that, as MF surgeries entail higher risks of preterm deliveries, potential benefits need to be balanced not only against the risks of the trial but also against the risks of prematurity.^26^

When it comes to the pregnant person, Hendriks et al. explained that MFS trials generally do not produce direct medical benefit for the pregnant person.^16^ Chervenak and McCullough maintained that it can be ethically acceptable to conduct a MFS trial for fetal benefit if risks for the pregnant person are reasonable.^17^ Regardless, seven publications emphasized that maternal risks should be minimized.^2,3,16–19,26,27^

Seven publications advised to include psychosocial factors in the risks-benefits assessment.^3,16,21,23,25,26,28^ For example, Hendriks et al. (2020) maintained that if a condition is associated with psychosocial harm, such as being less likely to achieve higher education, alleviating it is a benefit.^16^ The biggest discussion, however, is related to psychosocial risk-benefit assessments for the pregnant person. According to included publications MFS trials can produce psychosocial benefits for the pregnant person.^16,25,26^ In particular, they say, having a healthy child would benefit parents and children alike.^16,26^ Hendriks et al. reported that fetal repair of myelomeningocele results in less personal strain and lower family and social impact than the postnatal repair.^16^ One publication elicited that there exist psychosocial benefits related to the decision itself, including pregnant persons’ recognition that they did everything possible for their child, relief from the stress and anxiety of waiting until after birth, and/or preventing the burden of pregnancy termination.^16^ However, few authors emphasized that these evaluations are subjective and should be done by pregnant people themselves.^3,16,21,25,28^ To this regard, Hendriks et al. warned that excluding psychosocial factors from risk-benefits assessment in research protocols might lead to ethics committees rejecting MFS trials because there is no benefit for the woman.^16^

Four publications reminded that psychosocial risks for the pregnant person should also be acknowledged and minimized.^3,16,26,28^ For example, the psychosocial harm resulting from having to live close to the hospital and far from their children for a prolonged period,^16^ or the risk of excessive hope that might lead pregnant people to undermine their own safety for the fetus.^3,28^ Because of that, these publications suggested prioritizing medical risk-benefit assessment^3^ as the psychosocial benefits alone are not sufficient to justify the notable medical risks of MFS trials.^16^

To harmonize all these different considerations, Hendriks et al. proposed to conduct three risks-benefits assessments.^16,25^ First, the overall risk–benefit ratio of the study, i.e., risks and benefits for the fetus and the pregnant person, and social value. Second, the risk–benefit ratio for the pregnant person. If the overall risk-benefit ratio is acceptable, then pregnant people should decide for themselves whether to participate in MFS trials as it is acceptable that autonomous adults accept a setback of their interest to benefit others as in the case of living organ donation. If the risks for the pregnant person are excessive, then the MFS trial is unacceptable regardless of fetal benefit. Third, the risk–benefit ratio for the fetus. If the risk-benefit ratio is only slightly unfavorable for the fetus, then the MFS trial could still be justifiable in light of social value, but if the risks are excessive, then the MFS trial is unjustifiable regardless of social value. Importantly, Hendriks et al. advised to assess risk and benefit in the context of available treatment. For example, higher risks are more justifiable when no effective treatment exists.^16^

#### Informed consent

Eleven publications highlighted that adequate informed consent can be difficult to obtain in MFS trials.^2,16,23,24,26,28–33^ That is firstly because of the scientific uncertainty and high-risks embedded in these trials.^28–30,32,33^ Further, parents are often shocked and anxious due to the diagnosis^28,33^ and most of them never had to take such a weighty decision with life-changing potentially irreversible consequences.^32^ Others warned that pregnant people are often under internal or external pressure to participate^2,16,23,24^ and to sacrifice themselves for their children.^16^

Finally, three publications explained that parents make decisions not only based on clinical facts but also on values, religious beliefs, predetermined beliefs about surgery, information available online, etc.^26,28,30^ De Bie et al. warned that clinicians themselves can be influenced by personal and professional convictions.^26^ According to two publications the problem is that standard informed consent procedures do not address these kinds of influences.^28,30^

Many possible solutions have been proposed in the included articles to improve counseling in MFS trials and address the above-mentioned challenges.

First, Bartlett and Bliton found that a procedure called “ethics review” helped parents decide whether to participate in the MOMS trial.^29,30^ This review is a meeting between parents and an ethics consultant after clinical counseling to clarify parents’ questions, process the clinical information, prepare them to confront families’ and communities’ questions, and reflect and elaborate on ethical issues.^28,30^

Second, counseling must be comprehensive and conducted by a competent physician,^2,4,24^ or by a third-neutral party.^32^ Two publications advise multidisciplinary counseling - including MF specialists, neonatologists, and other relevant professionals - to minimize professionals’ bias and over-directive counseling, while ensuring that all angles are represented and explored.^26,34^ Engels et al. found that web-based additional information were perceived as helpful from parents.^35^

Third, counseling must be non-directive.^2,23,31^ Pregnant patients must know that they are under no obligation to accept the trial and, if necessary, the family should be reminded that they have no right to make a decision on behalf of the pregnant person.^2,31^ One publication added that, parents confronted with MFS trials for non-lethal conditions must know that these people live satisfactory lives and that continuing the pregnancy without surgery is also an option.^23^ Chervenak and McCullough advised researchers to be neutral and non-directive also in the information provided online and in the trial advertisement as this can also influence parents.^2^

Fourth, according to De Bie et al. MFS counselors should learn from pediatric palliative care specialists the skill of communicating bad news effectively, straightforwardly, and empathically, while helping families understand the limitations of MFS.^26^

Finally, seven publications highlighted the importance of preventing therapeutic misconception^2–4,21,23,24,28^ by avoiding words like “treatment” or “therapy”, which might suggest that the MFS trial is curative,^2,4,24^ and by emphasizing that the outcomes of the trial are uncertain.^23,24,31^

### Research ethics issues and solutions

Several authors noted how innovative maternal-fetal surgeries were introduced in clinical practice as “innovative treatments” not needing a rigorous trial.^20,23,36^ According to these authors this compromised patients’ safety as well as the scientific validity of the data obtained. Hence, a rigorous randomized controlled trial approved by an ethics committee should be conducted before implementing any MFS, especially those for non-lethal conditions.^20,23,36,37^

To further protect research participants, Chervenak and McCullough proposed three ethical criteria to justify commencing a MFS trial.^2,4,17,18^ First, to prevent premature human investigation, a trial should only be commenced if animal data suggest that the experimental surgery has the potential to be life-saving or to prevent serious and irreversible harm to the fetus. Second, the trial must be designed in a way that minimizes risks of death and morbidity for the fetus. Third, the risks for the pregnant person, including risks for future pregnancies, must be low or manageable.

#### Randomization

According to the included literature, achieving randomization can be especially challenging in MFS trials for the following reasons.

The biggest challenge to randomization identified by the literature is difficulties in recruitment.^7,21,33,38–44^ Randomized controlled trials require a large number of participants, whereas achieving a sizable sample has been proven challenging for MFS trials. For example, the MOMS trial and the TOTAL trial took longer than expected due to slow recruitment,^41,45^ and the PLUTO trial was interrupted due to lack of participants.^7^ Difficulties in recruitment are themselves a result of several factors. Included publications noted that these conditions are rare^7,38–40^ and often diagnosed only after birth.^7^ MFS trials have strict inclusion criteria, necessary to protect participants, but that further reduce the number of potential participants.^39,42^ On top of that, many parents choose termination of pregnancy.^7^ Another issue is that physicians and patients have their own personal conviction on whether prenatal or postnatal treatment is best.^7,21,33,39,43,44^ For example, Morris et al. found that women were reluctant to participate in the PLUTO trial either because they were pessimistic about the benefit of the surgery or because they wanted the prenatal surgery and they were unwilling to accept randomization, i.e., the possibility of being placed in the standard treatment group.^33^ Further, Chescheir and D’Alton reported that many clinicians did not refer eligible participants for prenatal closure of myelomeningocele because they were unaware of the MOMS trial.^39^

A second challenge to randomization reported in the literature are difficulties in standardization.^33,38,40,41,44,45^ Standardization is necessary but also challenging because each patient’s anatomy, physiology, and intraoperative course is unique,^38^ and because different countries have different standards of treatment.^41,44,45^ Another problem is the complexity of the conditions targeted by MFS trials. As Lally and Skarsgard explained “For a complex condition like CDH, which requires highly integrated multidisciplinary care (including surgery), the RCT, which generally targets one intervention at a time, is poorly suited to the creation of generalizable evidence which can be widely and safely implemented outside of the constrained trial environment”.^42, p.130]^ This means that MFS requires specialized multidisciplinary care that is not available in all hospitals.^40,41,45^ Finally, practical issues, such as the high costs of randomized controlled trials^38,44^ or lack of support for international collaborations,^33^ might also hinder randomization.

Possible solutions identified in the literature are: facilitating international recruitment^33^ and centralizing management and collaboration across centers,^38^ standardizing the experimental surgery but also the pre- and post-operative management,^38^ improving clinicians’ knowledge of MFS trials,^38^ and developing alternative models of/to randomization.^33^ Regarding the latter, Lally et al. proposed to develop a disease-specific registry: “a collection of standardized information about a group of patients who share a condition or experience that serves a predetermined scientific, clinical, or policy purpose.”^42, p.129]^ These registries would allow comparison of MFS trials outcomes with standard of care outcomes without classic randomization.^40^

#### Generalizability

Several publications explained that generalizability can be difficult to achieve in MFS trials due to different factors.^39,41,45–48^ First because of the strict inclusion criteria. These are necessary to ensure rigor, scientific validity, and patient protection, but they also hinder generalizability as the extent to which MFS trials data can be generalized to an expanded population is not always clear.^46–48^ For example, Marquart et al. observed that maternal obesity was an exclusion criterion of the MOMS trial because obesity is associated with complications in pregnancy and adult surgery.^46^ However, as obesity rates consistently increase it is not clear whether the outcomes of the MOMS trial can be generalized to this population.^46^ Similarly, Laskay et al. explained that including only people with an adequate support network, which was necessary to protect participants, combined with travel and follow-up requirements lead to a rather homogenous sample.^48^ Second, due to the lack of long-term pediatric and maternal data, including obstetric psychologic, gynecologic, and general health data, which is often a result of lack of funding.^39^ Finally, due to a lack of proper standard of care. Three publications explained that establishing a proper standard of care for MFS trials is challenging because different hospitals have different resources, practices, and variable experience in treating complex fetal conditions.^41,44,45^ Further, the standard of treatment against which MFS is compared can be questionable. For example, Villle explained that the experimental FETO procedure was compared to standard of care while most parents opted for termination of pregnancy.^41^ Similarly, two publications lamented that the TOTAL trial lasted so long that the experimental surgery outcomes are compared to outdated control procedures.^41,45^

#### Equipoise

There seems to be a significant agreement in the included literature that an MFS trial should only commence when there is equipoise,^2,4,31,49^ i.e., “a genuine uncertainty regarding benefits and risks of an investigational intervention as compared to the current best-proven therapy within the scientific community”.^50, p.177]^ However, many maintained that achieving equipoise can also be particularly challenging in MFS trials.^2–4,6,17,19–22,25,28,29,31,43,49–53^

First because equipoise as a concept rests on the idea that participants, like patients, are entitled to the best care available, as Rodrigues and Van Den Berg explained.^21^ However, a trial’s main aim is not providing the best care but producing generalizable knowledge. The therapeutic benefit is often uncertain at the beginning of the trial and it is to be proven within the trial.^21,52^ Hendriks et al. also elicited how the significance of MFS trials’ benefit is also difficult to determine as it correlates to the severity of the condition, which is often difficult to predict in utero.^52^

Second, authors explained that if we conceive the pregnant person and fetus as two distinct patients, equipoise will never be achieved for the pregnant person because the postnatal intervention will always be less risky and more beneficial than the in-utero surgery.^6,16,19,22,25^ There might be psychosocial benefit but, as previously mentioned, these are not substantial enough to justify the risks of the surgery.^3,6,21,52^ That is why, some authors believe that considering the pregnant person as the primary participant/patient would solve this conundrum as autonomous people are entitled to decide for themselves whether the risks of the trial are worth it.^6,19–21^ Equipoise for the fetus is also difficult to achieve as according to Hendriks et al. because some risks are unquantified and difficult to objectively assess, such as fetal loss or survival with severe disability.^52^

Third, some explained that equipoise might still be uncertain at the end of the trial.^28,29,37,50,51,53^ For example, Fisk et al. ^51^ found that the Eurofetus trial demonstrated that laser treatment is the best treatment for (almost) all stages of twin-to-twin transfusion syndrome, but equipoise is uncertain for the early treatment due to the high number of laser-related fetal losses.^51^ Vergote et al. found that a significant percentage of clinicians are still unsure of the risk-benefit ratio of FETO surgery for congenital diaphragmatic hernia,^50,53^ and Lyerly et al. found that many clinicians did not believe that in-utero repair of myelomeningocele was sufficiently validated.^37^ Similarly, two publications explained that MFS trials reduce but do not completely remove uncertainty. For example, although the MOMS trial was successful, some infants did not benefit from it.^28,29^ Meaning that individual parents and doctors are still confronted with the difficult question of whether the surgery is the best for this individual infant.^28,29^

To solve the uncertainties related to equipoise Chervenak and McCullough proposed three criteria to determine whether equipoise is reached. First, when the initial pre-trial data indicate that the experimental fetal intervention is reliably expected either to be lifesaving or to prevent serious harm. Second, that the experimental intervention involves the least amount of risks for the fetus among the possible interventions. Third, that the risks for the pregnant woman are reliably expected to be low or manageable.^2,4,17,31^

## Discussion

Our systematic review is based on 43 normative and empirical publications that offer a comprehensive and nuanced overview of the ethical issues raised by MFS trials. The analysis followed the rigorous and systematic QUAGOL methodology^14,15^ and was conducted in an interdisciplinary group. However, all articles originated from high-income western countries, mainly the USA (30/43), which might have affected the generalizability of the results. Interestingly, except for the discussion on whether the fetus should be considered as a participant, most of the ethical issues discussed were practical in nature and common to other trials that involve rare or uncommon conditions. Further, although there is disagreement on the moral status of the fetus, there seems to be a general agreement that the pregnant person’s has the right to decide whether to participate regardless of fetal benefit.^2,4,16–19^ In other terms, although there is a theoretical disagreement, everyone agrees on how to act in practice.

An overarching practical issue was whether clinicians should be allowed to conduct innovative treatments without a trial.^20,23,36^ It goes without saying that beneficence and non-maleficence should guide the whole trial.^54^ This means that we agree with included publications that a trial can only commence when equipoise is reached,^2,4,31,49^ and that clinicians should not be allowed to conduct innovative procedures, especially when invasive, outside the strictly regimented and controlled environment of a trial.^20,23,36,37^ Innovative procedure should only be tested on digital or animal models to ensure safety before the in-human trial. Other important issues were recruitment, standardization and contextualization of ethical reflection.

### Recruitment

Included publications elicited difficulties in recruitment that might result in delays or cancellations of MFS trials. Although these issues seem more technical than ethical, they entail relevant ethical ramifications. For example, the MOMS and TOTAL trials lasted notably longer than expected and the PLUTO trial was interrupted due to recruitment issues.^33^ Consequently, the trial results are based on control procedures that are now known as suboptimal, which affected the scientific validity of the data.^41,45^ Needless to say, delays and interruptions imply a waste of (public) resources. More importantly, delays and interruptions of trials due to technical issues mean that participants were pointlessly harmed.^55^ A trial inevitably entails risks as there is still a certain level of uncertainty on whether the experimental procedure is beneficial and on what the exact risks are. These uncertainties should be clarified within the trial, which is one of the goals of the trial itself.^21,52^ A trial also entails additional harms for participants. These are not only the harms related to the experimental surgery, but also those related to additional control procedures necessary to ensure a proper data set and participants’ safety, such as additional blood draws and long-term follow-ups.^56^ Risks and harms are justifiable if the trial benefits participants or produces valuable knowledge. However, if the trial is interrupted, like the PLUTO trial, or delayed to the point that data collected are outdated, like the MOMS trial, then participants have been pointlessly exposed to risk. This means that for these trials we need to develop creative ad hoc solutions that can prevent some of these issues, while still providing valuable knowledge and ensuring participants’ safety.

Another issue resulting from recruitment challenges is generalizability. Due to the practical difficulties in participating in the trial, such as high costs for participants, and the strict inclusion criteria, trial samples were rather homogeneous, which clearly hinders generalizability. This elicits a tension between the need to protect participants and the need to ensure scientific validity. For example, low socio-economic status was a common exclusion criterion in MFS trials established with the aim of avoiding undue influence (Wilpers et al. ^57^; Laskay et al. ^48^). MFS trials also excluded participants who did not have supporting person/network as participants in a vulnerable state are at higher risks (Wilpers et al. ^57^; Laskay et al. ^48^). This resulted in a homogeneous sample. Finding a solution for this conundrum goes beyond the scope of this article and we do not believe that there is an easy solution. However, we believe that these difficulties should be acknowledged and as much as possible addressed beforehand. For example, a parent support group could be created to support also those participants who do not have their own support network. This will allow to be more inclusive and it would potentially be beneficial for all participants.

### Standardization

A second recurrent practical issue that emerged from the included literature is standardization. A potential solution to ensure sufficient participants is to increase multicenter and international trials.^33^ To ensure scientific validity, it is important that the trial protocol is standardized across all participating centers and countries. However, experiences from the TOTAL trial demonstrated that standardizing the experimental protocol is not sufficient.^41,45^ For example, although the TOTAL trial protocol was standardized, the control treatment was not and substantially varied across countries, hindering, this way, the existence of a proper control group.^41,45^ Hence, the control treatment should also be standardized to ensure scientific validity. Further, lack of standardization in counseling can also be problematic as researchers’ and referring clinicians’ attitudes towards the trial can create undue influence. Researchers might be overly optimistic about their experimental procedure whereas referring clinicians could be overly pessimistic.^58^ To obtain true informed consent counseling needs to be at least partially standardized. Clearly, counseling needs to be tailored to each parent’s needs, but certain elements of the counseling should be at least similar in all counseling session. Particularly, the content of counseling should be consistent: all parents should receive complete information about the trial and the existing alternatives.^54^ Further, clinicians and researchers should be trained in conducting non-directive counseling to prevent (involuntarily) influencing parents toward a specific option.^58^

### Contextualization of ethical reflection

Taking a broader perspective, all these specific practical issues demonstrate that ethical reflection should be contextualized. Ethical reflection does not occur in a vacuum; rather, contextual factors hugely influence our ethical decision-making.^59^ For example, included publications elicited how contextual factors such as parents’ emotional status, practical difficulties in participation (e.g., lack of accommodation), and physicians’ attitudes toward the experimental surgery greatly influence whether parents will opt for trial, abortion, or standard of treatment.^24,26,28,32,48,60^ Contextual factors can also lead people to perceive and evaluate the same facts differently and, consequently, to reach different decisions. For example, while some emphasized that MFS trials benefit mainly the fetus and that counselors must stress the alternative to the trial,^6,21,22,52^ a study on parents’ decision-making found that many parents saw the trial as the only option.^61^

The fact that we reach different ethical evaluations based on contextual factors is not an issue per se, but it can become an issue if it is not acknowledged and addressed appropriately. For example, the fact that extra trial-related costs like travel and accommodation are not always covered might affect parents’ ability to participate and, in some cases, hinder sample diversity.^48,57,60^ We agree with Lyerly et al. that this issues should be acknowledged and addressed in future trials.^60^ To make another example, as previously mentioned, it is important to acknowledge that parents’ decisions are influenced by several factors. Hence, it is important to address these factors in the counseling and to leave parents enough time and space to reflect and decide, similar to what Bartlett and Bliton described as “ethical review”.^30,62^ A crucial part of this process is to empower the pregnant person to decide whether to participate in a trial that, as any trial, will inevitably entail risks and uncertainty. We believe that to achieve that counselors should be honestly and openly acknowledge the uncertainties related to the trial. Clinicians also need to help the pregnant person understand if participating the trial is the best option for them personally. Counselors should help the pregnant person to identify the most important values for them and their pregnancy rather than imposing their own values onto the pregnant person and develop a care plan that reflects the patient’s values. For example, if the person is extremely risk-averse a trial might not be the best option for them. To the opposite, if they want to ensure that they did “everything possible” than the trial might be the right option.

It is equally important to acknowledge that clinicians’ counseling can also be influenced by contextual factors.^26^ These can be clinicians’ own values, ensuring a successful and scientifically rigorous trial (e.g., need to ensure sufficient participants), or scientific facts (e.g., the trial might be too risky for this patient). When it comes to trial needs, these should be clarified and addressed beforehand. In other terms, the trial should be designed in a way that guarantees scientific validity while protecting participants, for example by using preexisting datasets instead of a RCT to ensure a scientifically valid control group while respecting parents request to receive the experimental procedure.^55^ Researchers should also be aware that the benefit of participants, including being free to make their own choice, always supersede the interests of research. When it comes to counselors personal biases, this could be addressed, for example, by non-directive counseling. This means that counselors prioritize the values of the pregnant person, as described above, and reassures the pregnant person that there are no good or bad choices in this context. The counselor can also openly admit their own values and biases. As long as they are transparent and communicated respectfully this could enrich the discussion. Finally, to avoid possible researchers’ excessive optimism toward the experimental treatment, involving a neutral counselor, as was proposed for similar trials.^32,55,63^

## Conclusions

Our results show that the main ethical issues raised by MFS trials are practical issues related to trial design and conduction. Particularly important are difficulties related to achieving standardization and adequate samples without huge delays. These issues have relevant ethical ramification as difficulties in recruitment and standardization might affect scientific validity and pointlessly harm participants. Hence, we need to develop creative solutions that can prevent some of these issues, while still providing valuable knowledge and ensuring participants’ safety. To do so, we need to involve all the relevant stakeholders (e.g., patient organizations, clinicians, scientific organizations) to better explore these issues from all the different perspectives and to develop solutions that truly answer the needs of all the parties involved.

## Supplementary information

Additional file 1

Checklist

## Data Availability

Available at request. To request original material, contact the corresponding author at alice.cavolo@ibme.uzh.ch.
