# Supplementary material for: Ethical challenges in conducting maternal-fetal surgery trials. A systematic review
Source: Pediatr Res. 2024 Dec 5;98(2):479–90. doi: 10.1038/s41390-024-03734-y (PMC12454111; doi:10.1038/s41390-024-03734-y)
Supplement: Supplementary file 1 — Additional file 1 [file 41390_2024_3734_MOESM1_ESM.pdf]

Additional file 1. Overview of Bibliographic Databases Searched, Search Strings Used, and Search Results of Articles Identified

Database	Group 1a: Maternal fetal (trials)	Group 1b: Maternal fetal	Group 1c: Trials	Results<br>(1a OR(1b AND 1c)) lim. English<br>18/01/2024
**Pubmed**<br>(via NCBI, including MEDLINE)	"fetal trial"[tiab:~2] OR "fetal trials"[tiab:~2] OR "fetal RCT"[tiab:~0] OR "maternal fetal medicine"[tiab] OR "maternal foetal medicine"[tiab] OR "total trial"[tiab] OR "Management of Myelomeningocele Study"[tiab] OR “MOMS trial”[tiab:~2] OR “MOMS study”[tiab:~2] OR “LUTO trial”[tiab:~0] OR “LUTO study”[tiab:~0] OR “TOTAL study”[ti] OR “Tracheal Occlusion To Accelerate Lung growth-trial”[tiab:~0] OR "foetal trial"[tiab:~0] OR "foetal trials"[tiab:~0] OR "foetal RCT"[tiab:~0] OR "maternal fetal surgery"[tiab] OR "maternal foetal surgery"[tiab]	"Fetal Therapies"[Mesh:NoExp] OR ("Fetal Diseases"[Mesh:NoExp] AND 1982:2004[mhda]) OR "fetal therap*"[tiab] OR "foetal therap*"[tiab] OR "fetal surg*"[tiab] OR "foetal surg*"[tiab] OR "Fetoscopy"[Mesh] OR “fetoscop*”[tiab] OR “foetoscop*”[tiab] OR “FETO”[tiab] OR “prenatal surgery”[tiab:~2] OR “antenatal surgery”[tiab:~2] OR “Fetal lower urinary tract obstruction”[tiab] OR “LUTO”[tiab] OR “Myelomeningocele”[tiab] OR "Meningomyelocele"[tiab] OR "Meningomyelocele"[Mesh] OR "Hernias, Diaphragmatic, Congenital"[Mesh] OR "Congenital Diaphragmatic Hernia"[tiab] OR "Fetal Diagn Ther"[jour]	"Clinical Trials as Topic"[Mesh:NoExp] OR "Controlled Clinical Trials as Topic"[Mesh:NoExp] OR "RCT"[tiab] OR "Randomized Controlled Trials as Topic"[Mesh:NoExp] OR “randomized controlled trial”[tiab] OR “randomised controlled trial”[tiab] OR "Multicenter Studies as Topic"[Mesh] OR “multicentre study”[tiab] OR “multicenter study”[tiab] OR “Off-trial access”[tiab:~0] OR “Expanded trial access”[tiab:~0]	Hits: 3004
**Embase**<br>(via Embase.com)	'maternal fetal surgery'/exp OR 'maternal fetal surg*':ti,ab,kw OR 'maternal foetal surg*':ti,ab,kw OR ‘fetal NEAR/0 trial’ OR ‘fetal NEAR/0 trials’ OR ‘fetal NEAR/1 RCT’ OR ‘total trial’:ti,ab,kw OR ‘Management of Myelomeningocele Study’:ti,ab,kw OR ‘MOMS NEAR/0 trial’ OR ‘MOMS NEAR/0 study’ OR ‘Management of Myelomeningocele Study’:ti,ab,kw OR ‘LUTO NEAR/0 trial’ OR ‘LUTO NEAR/0 study’ OR ‘TOTAL study’:ti OR ‘Tracheal Occlusion To Accelerate Lung growth-trial’:ti,ab,kw OR ‘foetal NEAR/0	'fetal therapy'/exp OR ‘fetus disease’/mj OR ‘fetus disease’:ti,ab,kw OR ‘fetal diseases’:ti,ab,kw OR ‘fetal surg*’:ti,ab,kw OR ‘foetal surg*’:ti,ab,kw OR ‘fetoscopy’/mj OR ‘Fetoscopy’:ti,ab,kw OR ‘fetoscop*’:ti,ab,kw OR ‘foetoscop*’:ti,ab,kw OR ‘FETO’:ti,ab,kw OR ‘prenatal surgery’:ti,ab,kw OR ‘antenatal NEAR/3 surgery’ OR ‘Fetal lower urinary tract obstruction’:ti,ab,kw OR ‘LUTO’:ti,ab,kw OR ‘Myelomeningocele’/mj OR ‘Meningomyelocele’:ti,ab,kw OR	'clinical trial (topic)'/de OR 'controlled clinical trial (topic)'/de OR ‘randomized NEAR/3 controlled NEAR/3 trial’ 'multicenter study (topic)'/exp OR 'RCT':ti,ab,kw OR 'multicenter stud*':ti,ab,kw OR 'multicentre stud*':ti,ab,kw OR 'multi center stud*':ti,ab,kw OR 'multi centre stud*':ti,ab,kw OR ‘off trial access’:ti,ab,kw OR ‘expanded trial access’:ti,ab,kw OR ‘trial’:ti,ab,kw	Hits: 1317

	trial' OR 'foetal NEAR/0 trials' OR 'foetal NEAR/0 RCT'	'Congenital Diaphragmatic Hernia':ti,ab,kw		
**WOS Core collection** (Editions: SCI-EXPANDED; SSCI; AHCI; CPCI-S; CPCI-SSH; ESCI)	"maternal fetal surg*" OR "maternal foetal surg*" OR "fetal NEAR/3 trial" OR "fetal NEAR/0 trials" OR "fetal NEAR/0 RCT" OR "maternal fetal medicine" OR "maternal foetal medicine" OR "total NEAR/0 trial" OR "Management of Myelomeningocele Study" OR "MOMS NEAR/0 trial" OR "MOMS NEAR/0 study" OR "Management of Myelomeningocele Study" OR "LUTO NEAR/0 trial" OR "LUTO NEAR/0 study" OR "TOTAL NEAR/0 study" OR "Tracheal Occlusion To Accelerate Lung growth-trial" OR "foetal NEAR/0 trial" OR "foetal NEAR/0 trials" OR "foetal RCT"	"fetal therap*" OR "foetal therap*" OR "fetus disease" OR "fetal diseases" OR "fetal surg*" OR "foetal surg*" OR "fetoscopy" OR "fetoscop*" OR "foetoscop*" OR "FETO" OR "prenatal surgery" OR "antenatal NEAR/3 surgery" OR "Fetal lower urinary tract obstruction" OR "LUTO" OR "Myelomeningocele" OR "Congenital Diaphragmatic Hernia"	"clinical trial" OR "controlled clinical trial" OR "RCT" OR "multicenter stud*" OR "multicentre stud*" OR "off trial access" OR "expanded trial access" OR "trial"	Hits: 2615
**CINAHL** (via EBSCOhost)	(MH "Fetal Surgery") OR TI ("maternal fetal surg*" OR "maternal foetal surg*" OR "fetal -> N3 trial" OR "fetal -> N0 trials" OR "fetal -> N0 RCT" OR "maternal fetal medicine" OR "maternal foetal medicine" OR "total -> N0 trial" OR "Management of Myelomeningocele Study" OR "MOMS -> N0 trial" OR "MOMS -> N0 study" OR "Management of Myelomeningocele Study" OR "LUTO -> N0 trial" OR "LUTO -> N0 study" OR "TOTAL -> N0 study" OR "Tracheal Occlusion To Accelerate Lung growth-trial" OR "foetal -> N0 trial" OR "foetal -> N0 trials" OR "foetal RCT") OR AB ("maternal fetal surg*" OR "maternal foetal surg*" OR "fetal -> N3 trial" OR "fetal -> N0 trials" OR "fetal -> N0 RCT" OR "maternal fetal medicine" OR "maternal foetal	TI ("fetal therap*" OR "foetal therap*" OR "fetus disease" OR "fetal diseases" OR "fetal surg*" OR "foetal surg*" OR "fetoscopy" OR "fetoscop*" OR "foetoscop*" OR "FETO" OR "prenatal surgery" OR "antenatal -> N0 surgery" OR "Fetal lower urinary tract obstruction" OR "LUTO" OR "Myelomeningocele" OR "Congenital Diaphragmatic Hernia") OR AB ("fetal therap*" OR "foetal therap*" OR "fetus disease" OR "fetal diseases" OR "fetal surg*" OR "foetal surg*" OR "fetoscopy" OR "fetoscop*" OR "foetoscop*" OR "FETO" OR "prenatal surgery" OR "antenatal -> N0 surgery" OR "Fetal lower urinary tract obstruction" OR "LUTO" OR "Myelomeningocele" OR "Congenital Diaphragmatic Hernia")	TI ("clinical trial" OR "controlled clinical trial" OR "RCT" OR "multicenter stud*" OR "multicentre stud*" OR "off trial access" OR "expanded trial access" OR "trial") OR AB ("clinical trial" OR "controlled clinical trial" OR "RCT" OR "multicenter stud*" OR "multicentre stud*" OR "off trial access" OR "expanded trial access" OR "trial")	Hits: 1361

	medicine” OR “total -> N0 trial” OR “Management of Myelomeningocele Study” OR “MOMS -> N0 trial” OR “MOMS -> N0 study” OR “Management of Myelomeningocele Study” OR “LUTO -> N0 trial” OR “LUTO -> N0 study” OR “TOTAL -> N0 study” OR “Tracheal Occlusion To Accelerate Lung growth-trial” OR “foetal -> N0 trial” OR “foetal -> N0 trials” OR “foetal RCT”)			
**PhilPapers** (via Philpapers.com)	fetal|foetal|spina bifida|fetus|foetus|moms|luto		trial*|stud*|RCT*	Hits:56

Additional file 2. Data analysis and synthesis following the Qualitative Analysis Guide of Leuven

Step[a]	Description
Familiarization with the publications	Articles were repeatedly read to familiarise ourselves with the material.
Individual conceptual schemes	AC developed individual conceptual schemes of every article. The schemes summarise the concepts and arguments emerging from the included articles. The schemes were refined based on team discussion.
Overall conceptual scheme	AC merged the individual conceptual schemes into a single overall conceptual scheme and refined the scheme after team discussion. These scheme provided the structure for reporting the results.
Reporting of results	We reported the results based on the overall conceptual scheme.

[a]A back-and-forth movement between all steps was maintained during the whole process to avoid overlooking relevant nuances and to ensure that we preserved the original meaning of the included publications.
